# Number of examined lymph nodes and overall survival in intrahepatic cholangiocarcinoma: a population-based study suggesting a staging benefit

**DOI:** 10.3389/fonc.2026.1925253

**Published:** 2026-09-28

**Authors:** Xuchang He, Wenhui An, Yangwei Chen, Shuang Wang, Yan He, En Luo

**Affiliations:** 1 Department of Hepatobiliary Surgery, The Fifth People’s Hospital Affiliated to Chengdu University of Traditional Chinese Medicine, Chengdu, China; 2 Critical Care Medicine, The Fifth People’s Hospital Affiliated to Chengdu University of Traditional Chinese Medicine, Chengdu, China; 3 Respiratory and Critical Care Medicine, West China Hospital, Sichuan University, Chengdu, China

**Keywords:** intrahepatic cholangiocarcinoma, lymph node yield, nodal staging, overall survival, SEER database, stage migration

## Abstract

**Background:**

The prognostic role of lymph node yield (LNY) in intrahepatic cholangiocarcinoma (ICC) remains debated. We aimed to clarify the role of LNY by examining its association with nodal detection and overall survival (OS).

**Methods:**

We identified patients with surgically resected ICC (2004–2022) from the SEER database. Multivariable Cox models and inverse probability of treatment weighting (IPTW) stratified by nodal status were used to evaluate the association between LNY (≥6 vs <6) and OS. Logistic regression examined predictors of nodal metastasis.

**Results:**

LNY ≥6 was not independently associated with OS (HR 0.88, 95% CI 0.71–1.08; P = 0.209). Nodal metastasis (HR 2.29), advanced T category, larger tumor size, poor differentiation, male sex, and lack of chemotherapy were independently associated with worse OS. Notably, multiple tumors were associated with better OS than single tumors (HR 0.79), likely reflecting selection bias. After IPTW, LNY remained non-significant in both the N0 (HR 0.81, 95% CI 0.59–1.11) and N1 (HR 0.95, 95% CI 0.70–1.29) subgroups. Higher LNY was strongly associated with detecting nodal metastasis (OR 4.06, 95% CI 2.99–5.52; P < 0.001).

**Conclusions:**

These findings suggest that nodal evaluation in resected ICC primarily improves staging accuracy, and are compatible with—though not directly confirmatory of—the stage-migration hypothesis. Adequate nodal assessment remains important for accurate prognostication, although a modest therapeutic effect of lymphadenectomy cannot be entirely excluded.

## 1 Background

Intrahepatic cholangiocarcinoma (ICC) is a rare and aggressive primary liver malignancy, with an incidence of approximately 1.2 cases per 100,000 person-years (1). Surgical resection remains the only potentially curative treatment; however, long-term outcomes are poor due to late-stage diagnosis, with a median survival of approximately 28 months and a 5-year overall survival (OS) rate of around 30%. As ICC accounts for only 10–15% of primary liver malignancies, high-level evidence to guide clinical management remains limited (2).

The role of lymph node yield (LNY) in ICC is particularly controversial. Lymph node metastasis and advanced TNM stage are well-established adverse prognostic factors, and current AJCC guidelines recommend the examination of at least six lymph nodes to ensure adequate nodal staging (3). However, recent meta-analyses have not demonstrated a clear survival benefit of higher LNY after resection (4). While LNY provides essential pathological information for accurate staging and prognostic stratification, its therapeutic value remains debated. Notably, several recent studies have suggested a potential survival benefit of higher LNY in selected patients, particularly among those with clinically node-negative disease or when an adequate number of lymph nodes are examined (5–7), whereas other retrospective analyses have failed to confirm this association and raised concerns regarding operative morbidity (8).

These inconsistencies may be partly explained by the substantial biological heterogeneity of ICC, variable lymphatic drainage patterns, and differences in surgical and pathological practices across institutions (9, 27). In the absence of robust prospective evidence, population-based analyses using the SEER database have provided complementary insights, although prior studies have yielded heterogeneous results, often without stratification by nodal status or adequate adjustment for treatment-related confounders (10–15).

Given these uncertainties, we conducted a population-based study using the SEER database to evaluate the association between LNY and OS in resected ICC. By separately analyzing node-negative and node-positive patients and integrating survival analyses with nodal detection, we aimed to distinguish the staging contribution of LNY from any apparent survival association and to clarify whether observed differences are more consistent with treatment effect or stage migration.

## 2 Methods

### 2.1 Data sources and study cohort

An analysis was performed using nationally representative data from the Surveillance, Epidemiology and End Results (SEER) database. SEER consists of 18 local and regional cancer registries, representing approximately 26% of the population in the USA (8). Patients diagnosed with intrahepatic cholangiocarcinoma (ICC) between 2004 and 2022 were identified from the SEER database. ICC was defined according to the International Classification of Diseases for Oncology, Third Edition (ICD-O-3), based on primary tumor site and histological codes. Patients were included if they underwent curative-intent surgical resection and had available information on lymph node evaluation and survival outcomes (**Supplementary Tables S1A, S1B**). Using this algorithm, 2890 patients with ICC were identified. Patients who did not undergo surgery, as well as patients with no data on lymph node examination or who had missing data on the pathologic examination of lymph nodes, were excluded (**Figure 1**). In turn, 1532 ICC patients who underwent curative-intent surgery who had complete data were included in the analytic cohort. This observational study was reported in accordance with the Strengthening the Reporting of Observational Studies in Epidemiology (STROBE) and the Reporting of studies Conducted using Observational Routinely-collected health Data (RECORD) guidelines.

**Figure 1:**
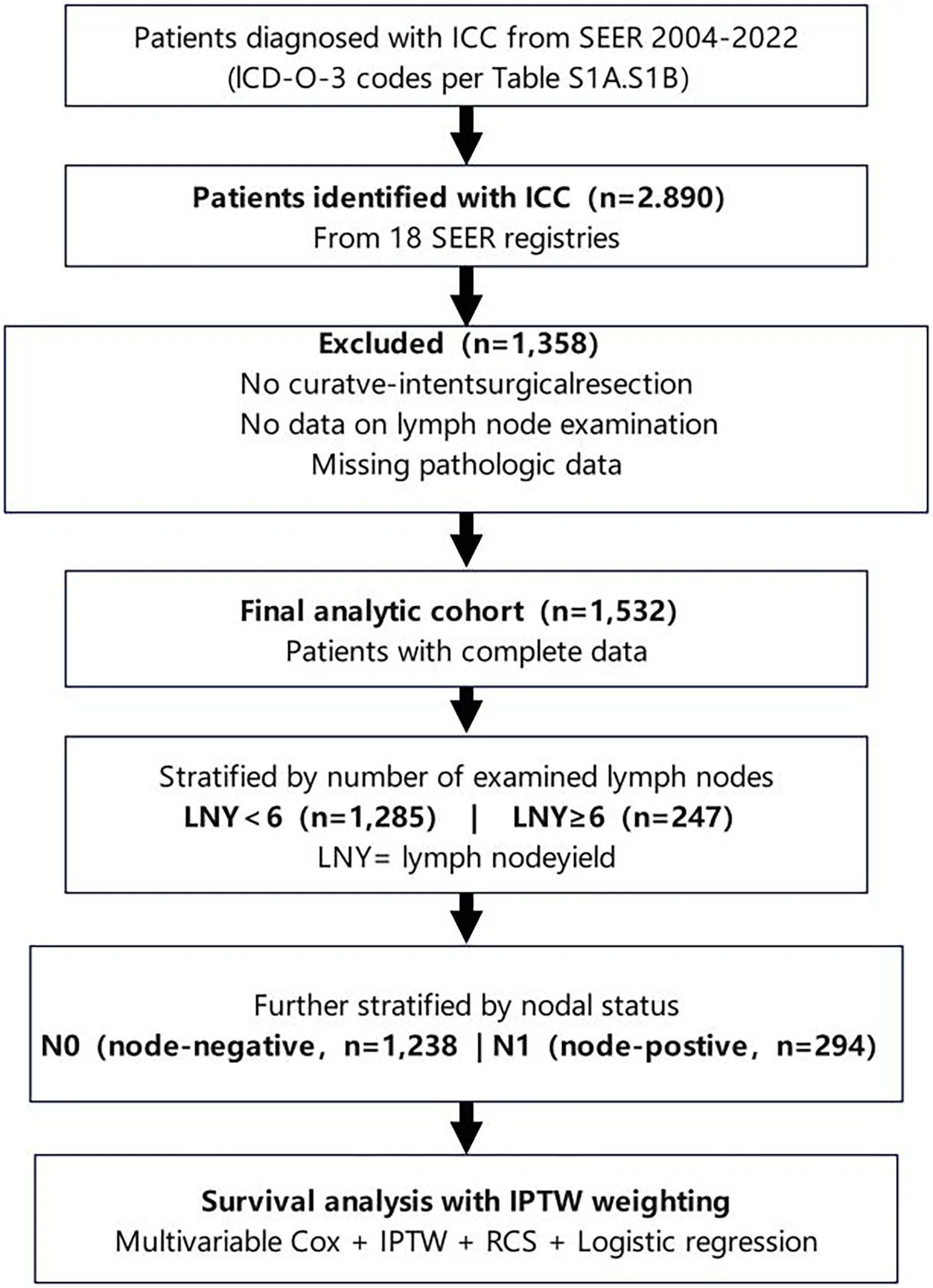
Flowchart of patient selection from the SEER database.

### 2.2 Variables and definitions

Demographic variables included age at diagnosis, sex, and race. Tumor-related variables included tumor size, tumor number, histological grade, and American Joint Committee on Cancer (AJCC) stage (3). Because the AJCC staging system was revised during the study period (6th edition for diagnoses 2004–2015, 7th edition for 2016–2017, and 8th edition from 2018 onward), and the boundaries between stage I–II and stage III–IV remained stable across editions, AJCC stage was dichotomized as stage I–II vs stage III–IV for descriptive purposes. Nodal status was classified as node-negative (N0) or node-positive (N1) according to the AJCC-derived N category in the SEER database, which integrates pathological and clinical (imaging) assessment; patients with no examined nodes were classified as N0 in the absence of recorded nodal positivity. Lymph node yield (LNY) was defined by the number of examined lymph nodes and categorized as <6 or ≥6 nodes, consistent with current AJCC recommendations (at least six examined nodes). AJCC stage was excluded from regression models due to collinearity with T and N stage; T category (T3–4 vs T1–2) and N stage (N1 vs N0) were included instead. Overall survival (OS) was defined as all-cause mortality.

### 2.3 Survival analysis

Overall survival (OS) was estimated using the Kaplan–Meier method, and differences between groups were compared using the log-rank test. Multivariable Cox proportional hazards regression models were constructed to identify independent prognostic factors associated with OS in the overall cohort. To further explore the prognostic role of LNY, survival analyses were also performed stratified by nodal status (N0 and N1). Restricted cubic spline (RCS) analyses with 4 knots, treating lymph node count as a continuous variable, were performed to evaluate potential non-linear dose–response relationships in N0 and N1 subgroups.

### 2.4 Inverse probability of treatment weighting

To address potential confounding due to baseline imbalances between lymph node yield groups, inverse probability of treatment weighting (IPTW) was applied. Propensity scores were estimated using multivariable logistic regression models that included the following covariates: age (<60 vs ≥60 years), sex, tumor size (continuous in mm), tumor multiplicity (single vs multiple), T category (T3–4 vs T1–2), histological grade (III–IV vs I–II), year of diagnosis (2004–2010, 2011–2014, 2015–2018, 2019–2022), N stage (N1 vs N0),and chemotherapy (yes vs no). Separate propensity score models were fitted for N0 and N1 patients to account for differential treatment patterns.

Stabilized IPTW weights were calculated as: weight = P(Z = 1)/PS for treated patients (LNY ≥6) and weight = (1 − P(Z = 1))/(1 − PS) for untreated patients (LNY <6), where PS denotes the propensity score and P(Z = 1) denotes the marginal probability of LNY ≥6 in the respective subgroup. Covariate balance before and after weighting was assessed using standardized mean differences (SMD), with a threshold of |SMD| < 0.10 considered adequate balance. After IPTW, weighted Cox proportional hazards models were used to estimate the association between LNY ≥6 and OS in the N0 and N1 subgroups separately, with robust standard errors to account for weighting.

### 2.5 Sensitivity analyses

Several sensitivity analyses were performed to assess the robustness of the primary findings. First, an alternative threshold of ≥3 examined lymph nodes was evaluated, given that some recent studies have suggested this cut-off may be clinically relevant. Second, the primary analysis was repeated after excluding 596 patients with no lymph nodes examined to assess whether the inclusion of these patients diluted the observed association. Third, restricted cubic spline analyses with 4 knots treating lymph node count as a continuous variable were performed in N0 and N1 subgroups to evaluate dose–response relationships and potential non-linearity. Fourth, analyses were stratified by AJCC staging era (2004–2015 vs 2016–2022) to account for revisions of the AJCC staging system during the study period, with a formal interaction test between LNY and staging era.

### 2.6 Analysis of nodal metastasis detection

Multivariable logistic regression was used to evaluate whether a higher number of examined lymph nodes was associated with nodal positivity (N1 vs N0), after adjustment for tumor burden and other clinically relevant factors, including age, sex, tumor size, T category, histological grade, tumor multiplicity, chemotherapy, and year of diagnosis. Chemotherapy was included as an adjustment covariate given its clinical relevance to treatment selection; it was not a predictor of interest, and its association with nodal positivity should be interpreted as exploratory. Tumor size was modeled as a continuous variable in millimeters in regression analyses.

### 2.7 Statistical analysis

All statistical analyses were conducted using R software (version 4.3.3). Survival analyses were performed using the survival and survminer packages. Forest plots were created using the forestplot package. Propensity score estimation and weighting were conducted using the MatchIt and WeightIt packages. Restricted cubic spline analyses were performed using the rms package. All statistical tests were two-sided, and a P value less than 0.05 was considered statistically significant.

## 3 Results

### 3.1 Baseline characteristics according to the extent of lymph node evaluation

A total of 1,532 patients with intrahepatic cholangiocarcinoma who underwent surgical resection were included in the analysis. Patients were stratified according to lymph node yield (LNY) into the LNY <6 group (n = 1,285) and the LNY ≥6 group (n = 247).Comparisons of baseline characteristics between the two groups are summarized in **Table 1**.

**Table 1:** Baseline characteristics of patients with resected intrahepatic cholangiocarcinoma, stratified by number of examined lymph nodes ***(<6 vs ≥6)***.

Variable	Category	Overall	LNY <6 (n=1285)	LNY ≥6 (n=247)	P
Age		1532 (100%)	1285 (100%)	247 (100%)	0.051
	<60	439 (28.7%)	355 (27.6%)	84 (34.0%)	
	≥60	1093 (71.3%)	930 (72.4%)	163 (66.0%)	
Gender		1532 (100%)	1285 (100%)	247 (100%)	0.133
	Female	817 (53.3%)	674 (52.5%)	143 (57.9%)	
	Male	715 (46.7%)	611 (47.5%)	104 (42.1%)	
Race		1532 (100%)	1285 (100%)	247 (100%)	0.671
	Black	107 (7.0%)	87 (6.8%)	20 (8.1%)	
	Other	221 (14.4%)	189 (14.7%)	32 (13.0%)	
	Unknown	5 (0.3%)	4 (0.3%)	1 (0.4%)	
	White	1199 (78.3%)	1005 (78.2%)	194 (78.5%)	
T stage		1532 (100%)	1285 (100%)	247 (100%)	0.001
	T1+T2	1238 (80.8%)	1058 (82.3%)	180 (72.9%)	
	T3+T4	294 (19.2%)	227 (17.7%)	67 (27.1%)	
AJCC stage		1532 (100%)	1285 (100%)	247 (100%)	0.000
	I+II	1038 (67.8%)	924 (71.9%)	114 (46.2%)	
	III+IV	494 (32.2%)	361 (28.1%)	133 (53.8%)	
N stage		1532 (100%)	1285 (100%)	247 (100%)	0.000
	N0	1238 (80.8%)	1096 (85.3%)	142 (57.5%)	
	N1	294 (19.2%)	189 (14.7%)	105 (42.5%)	
Tumor number		1532 (100%)	1285 (100%)	247 (100%)	0.184
	Multiple	467 (30.5%)	401 (31.2%)	66 (26.7%)	
	Single	1065 (69.5%)	884 (68.8%)	181 (73.3%)	
Grade		1532 (100%)	1285 (100%)	247 (100%)	0.890
	I+II	1057 (69.0%)	888 (69.1%)	169 (68.4%)	
	III+IV	475 (31.0%)	397 (30.9%)	78 (31.6%)	
Chemotherapy		1532 (100%)	1285 (100%)	247 (100%)	0.003
	No	733 (47.8%)	637 (49.6%)	96 (38.9%)	
	Yes	799 (52.2%)	648 (50.4%)	151 (61.1%)	
Radiation		1532 (100%)	1285 (100%)	247 (100%)	0.031
	No	1333 (87.0%)	1129 (87.9%)	204 (82.6%)	
	Yes	199 (13.0%)	156 (12.1%)	43 (17.4%)	

Significant differences were observed between the two groups in terms of tumor stage and nodal status. Patients in the LNY ≥6 group more frequently presented with advanced T category (T3–4: 27.1% vs. 17.7%, P = 0.001) and higher AJCC stage (III–IV: 53.8% vs. 28.1%, P < 0.001). The proportion of node-positive disease was substantially higher in the LNY ≥6 group compared with the LNY <6 group (42.5% vs. 14.7%, P < 0.001). Other demographic and tumor-related variables, including age, tumor size, tumor number, histological grade, chemotherapy and radiotherapy, were comparable between groups (all P > 0.05);however, patients in the LNY ≥6 group more frequently received chemotherapy (61.1% vs. 50.4%, P = 0.003) and radiotherapy (17.4% vs. 12.1%, P = 0.031).These findings suggest that more extensive lymph node evaluation was more commonly performed in patients with advanced disease features.

### 3.2 Multivariable Cox regression analysis

Multivariable Cox proportional hazards regression analysis was performed to identify independent prognostic factors for overall survival in the overall cohort (**Figure 2**). After adjustment for demographic, tumor-related, and treatment variables, LNY ≥6 was not independently associated with OS (HR 0.88, 95% CI 0.71–1.08; P = 0.209). N stage remained the strongest prognostic factor (N1 vs N0: HR 2.29, 95% CI 1.92–2.73; P < 0.001), followed by T stage (T3–4 vs T1–2: HR 1.60, 95% CI 1.35–1.90; P < 0.001) and chemotherapy (HR 0.79, 95% CI 0.67–0.92; P = 0.002). Other factors independently associated with worse OS included higher tumor grade (HR 1.36, 95% CI 1.17–1.58), larger tumor size (per 1 mm: HR 1.004, 95% CI 1.002–1.006), and male sex (HR 1.22, 95% CI 1.06–1.41). Of note, the overall model indicated that single tumors were associated with worse OS compared with multiple tumors (HR 1.27, 95% CI 1.08–1.50; P = 0.004). This counterintuitive finding likely reflects strong selection bias, whereby patients with multiple tumors who underwent surgical resection may represent a highly selected subgroup with more favorable underlying biology.

**Figure 2:**
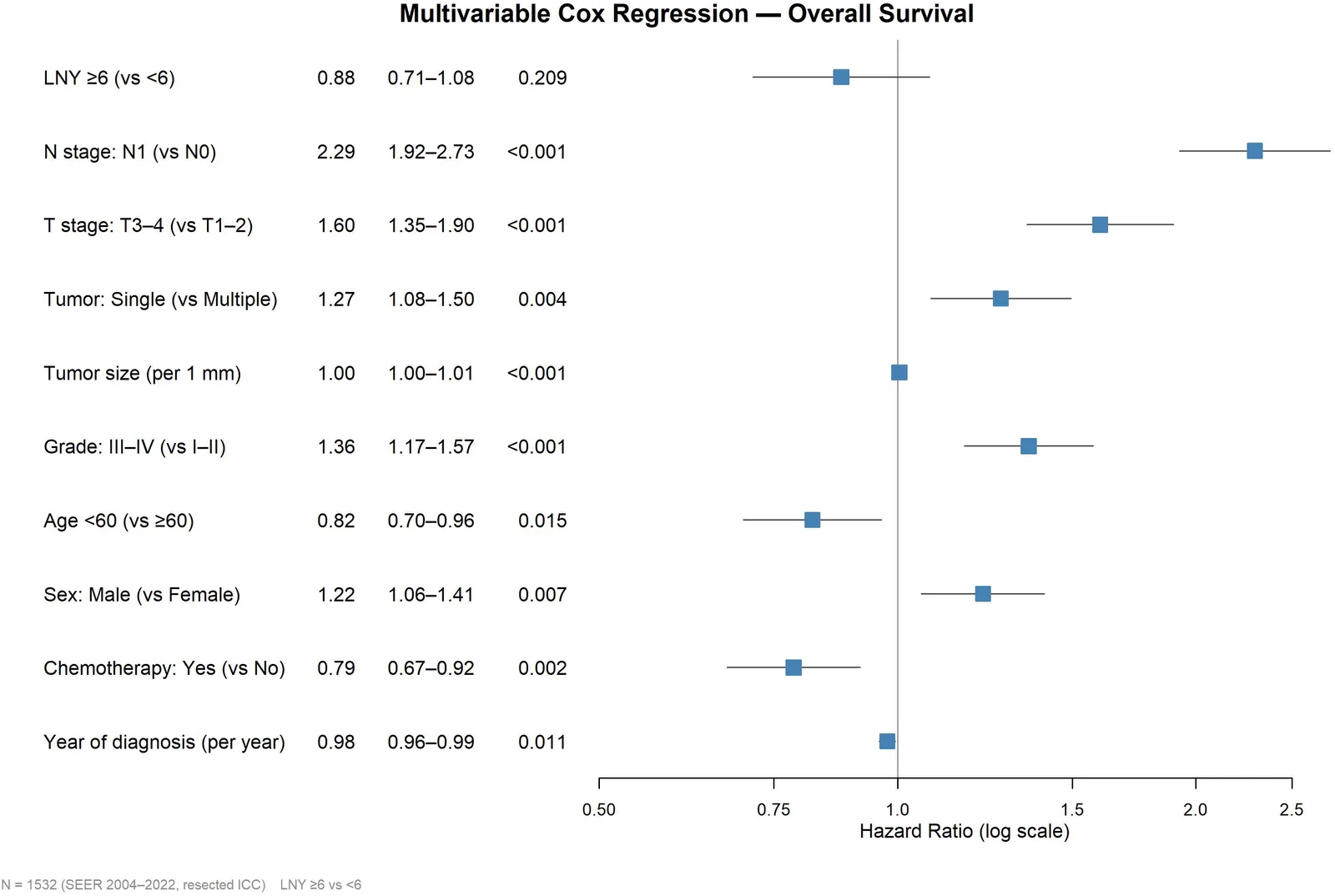
Multivariable Cox proportional hazards regression analysis of factors associated with overall survival in the overall cohort. Forest plot showing hazard ratios and 95% confidence intervals.

### 3.3 Association between lymph node yield and nodal status

To explore factors associated with nodal positivity, multivariable logistic regression analysis was performed (**Table 2**). After adjustment for tumor burden and other clinically relevant variables, LNY ≥6 was independently associated with nodal positivity (odds ratio [OR], 4.06; 95% CI, 2.99–5.52; P < 0.001).

**Table 2:** Multivariable logistic regression for predictors of nodal metastasis (N1 vs N0).

Variable	OR	95% CI		P value
LNY ≥6 (vs <6)	4.06	2.99	5.52	<0.001
Tumor size (per 1 mm increase)	1.00	1.00	1.01	0.074
T stage T3–4 (vs T1–2)	2.07	1.51	2.82	<0.001
Grade III–IV (vs I–II)	1.29	0.97	1.72	0.077
Age <60 (vs ≥60)	1.13	0.84	1.52	0.418
Male (vs Female)	1.09	0.83	1.43	0.522
Multiple tumors (vs Single)	0.96	0.70	1.30	0.780
Chemotherapy (Yes vs No)	1.68	1.26	2.23	<0.001
Later year of diagnosis	0.99	0.96	1.02	0.474

In addition, advanced T category (T3–4 vs. T1–2; OR, 2.07; 95% CI, 1.51–2.82; P < 0.001) was significantly associated with nodal positivity, whereas larger tumor size (per 1-mm increase; OR, 1.00; 95% CI, 1.00–1.01; P = 0.074) and poor differentiation (grade III–IV vs. I–II; OR, 1.29; 95% CI, 0.97–1.72; P = 0.077) did not reach statistical significance. Age, sex, and tumor multiplicity were not independently associated with lymph node status. These results indicate that higher lymph node yield was correlated with the identification of node-positive disease rather than patient-related demographic factors.

### 3.4 Survival analysis stratified by nodal status

Kaplan–Meier survival analyses were performed to evaluate the association between LNY extent and survival outcomes in N0 and N1 subgroups, both before and after IPTW adjustment (**Figures 3**, **4**). In the unadjusted cohort, no significant difference in survival was observed between patients with LNY <6 and ≥6 in either nodal subgroup.

**Figure 3:**
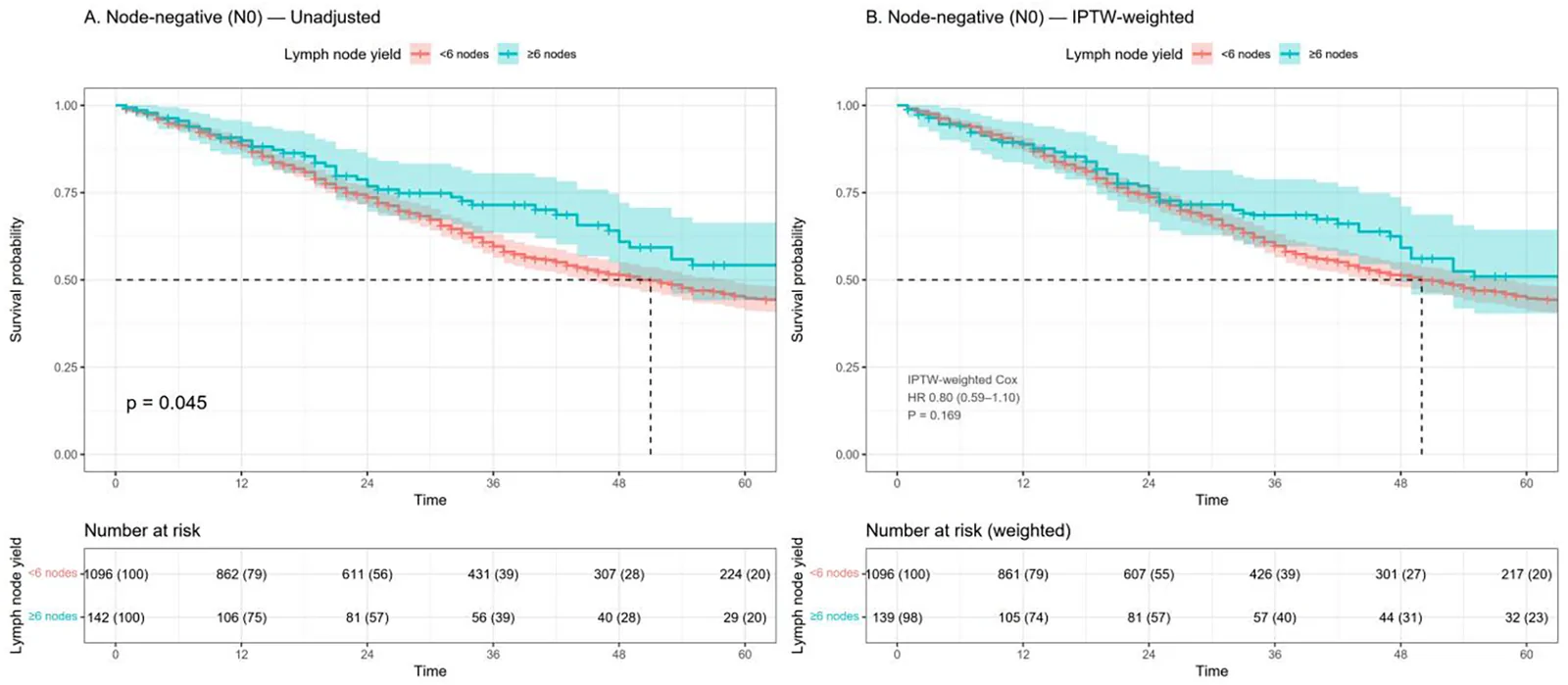
Overall survival (OS) in node-negative (N0) patients by number of examined lymph nodes (<6 vs ≥6). **(A)** Unadjusted Kaplan–Meier curves with log-rank test. **(B)** IPTW-weighted survival curves. Dashed lines indicate median survival. Numbers at risk are shown below each panel.

**Figure 4:**
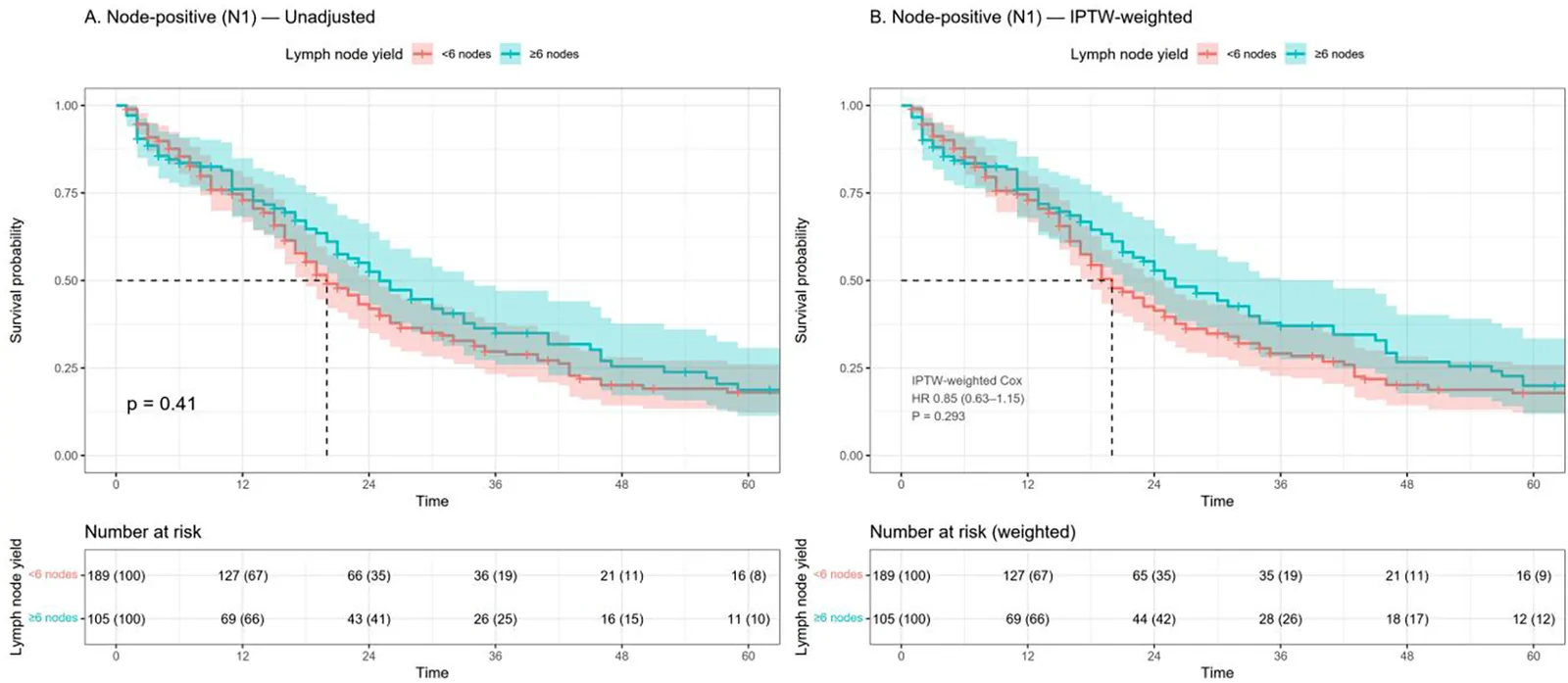
Overall survival (OS) in node-positive (N1) patients by number of examined lymph nodes (<6 vs ≥6), **(A)** Unadjusted Kaplan–Meier curves with log-rank test. **(B)** IPTW-weighted survival curves. Dashed lines indicate median survival. Numbers at risk are shown below each panel.

After IPTW adjustment, covariate balance between LNY groups was achieved, as demonstrated by standardized mean differences within acceptable thresholds (**Figure 5**; **Table 3**). In the IPTW-weighted Cox proportional hazards model for N0 patients, LNY ≥6 was not independently associated with improved survival (HR, 0.81; 95% CI, 0.59–1.11; P = 0.183). Younger age, female sex, smaller tumor size, lower T category, lower histological grade, and later year of diagnosis were independently associated with better survival (**Table 4**).

**Figure 5:**
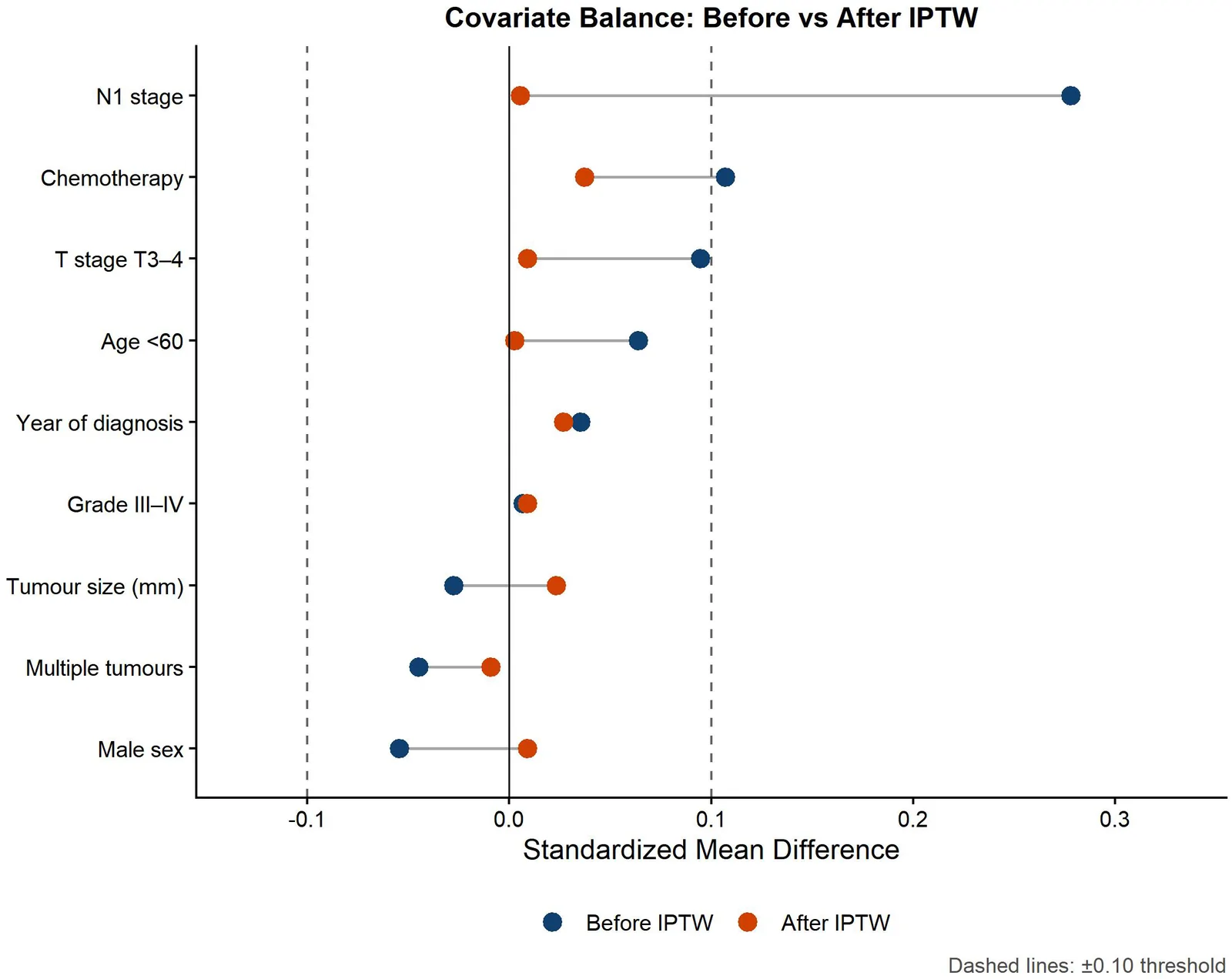
Love plot showing covariate balance before and after IPTW (standardized mean differences; threshold ±0.10).

**Table 3:** Baseline characteristics before and after IPTW.

Variable	Before IPTW LNY <6	Before IPTW LNY ≥6	SMD	After IPTW LNY <6	After IPTW LNY ≥6	SMD
Age <60 (%)	27.6%	34.0%	0.064	28.7%	28.9%	0.003
Male (%)	47.5%	42.1%	-0.054	46.6%	47.5%	0.009
T stage T3-4 (%)	17.7%	27.1%	0.095	19.1%	20.0%	0.009
Tumor size (mm, mean)	54.4	53.6	-0.028	54.3	55.1	0.023
Grade III-IV (%)	30.9%	31.6%	0.007	31.0%	31.9%	0.009
Multiple tumors (%)	31.2%	26.7%	-0.045	30.5%	29.5%	-0.009
Chemotherapy (%)	50.4%	61.1%	0.107	52.4%	56.1%	0.037
Year of diagnosis (mean)	2016.0	2016.5	0.119	2016.1	2016.4	0.064
N1 stage (%)	14.7%	42.5%	0.278	19.3%	19.8%	0.005

**Table 4:** IPTW-weighted multivariable Cox proportional hazards model for OS in node-negative (N0) patients.

Variable	HR	95% CI		P value
LNY ≥6 (vs <6)	0.81	0.59	1.11	0.183
Chemotherapy (Yes vs No)	0.91	0.76	1.09	0.294
Age <60 (vs ≥60)	0.77	0.63	0.94	0.010
Male (vs Female)	1.27	1.07	1.51	0.007
Tumor size (per 1 mm)	1.00	1.00	1.01	0.009
Multiple tumors (vs Single)	0.77	0.64	0.93	0.008
T stage T3–4 (vs T1–2)	1.79	1.44	2.23	<0.001
Grade III–IV (vs I–II)	1.42	1.18	1.72	<0.001
Later year of diagnosis	0.97	0.95	0.99	0.004

Similarly, in the N1 subgroup, IPTW-weighted Cox analysis demonstrated no significant association between LNY ≥6 and survival (HR, 0.95; 95% CI, 0.70–1.29; P = 0.753). In contrast, receipt of chemotherapy was independently associated with improved survival (HR, 0.45; 95% CI, 0.33–0.65; P < 0.001), whereas other clinicopathological variables did not reach statistical significance (**Table 5**).

**Table 5:** IPTW-weighted multivariable Cox proportional hazards model for OS in node-positive (N1) patients.

Variable	HR	95% CI		P value
LNY ≥6 (vs <6)	0.95	0.70	1.29	0.753
Chemotherapy (Yes vs No)	0.45	0.32	0.63	<0.001
Age <60 (vs ≥60)	1.07	0.79	1.45	0.649
Male (vs Female)	1.08	0.80	1.46	0.621
Tumor size (per 1 mm)	1.00	1.00	1.01	0.192
Multiple tumors (vs Single)	0.82	0.55	1.21	0.320
T stage T3–4 (vs T1–2)	1.31	0.96	1.80	0.092
Grade III–IV (vs I–II)	1.35	0.99	1.83	0.055
Later year of diagnosis	1.01	0.97	1.05	0.667

### 3.5 Restricted cubic spline analysis

To further characterize the dose–response relationship, restricted cubic spline (RCS) analyses with 4 knots were performed, treating lymph node count as a continuous variable (**Figure 6**). In N0 patients, the RCS revealed a monotonic decrease in the hazard of death with increasing node counts (overall P = 0.078), with formal testing for non-linearity yielding a non-significant result (P = 0.266), indicating an approximately log-linear relationship (**Figure 6A**).In N1 patients, the RCS showed a flat relationship with no significant association (overall P = 0.372; non-linearity P = 0.615), consistent with the primary analysis (**Figure 6B**).

**Figure 6:**
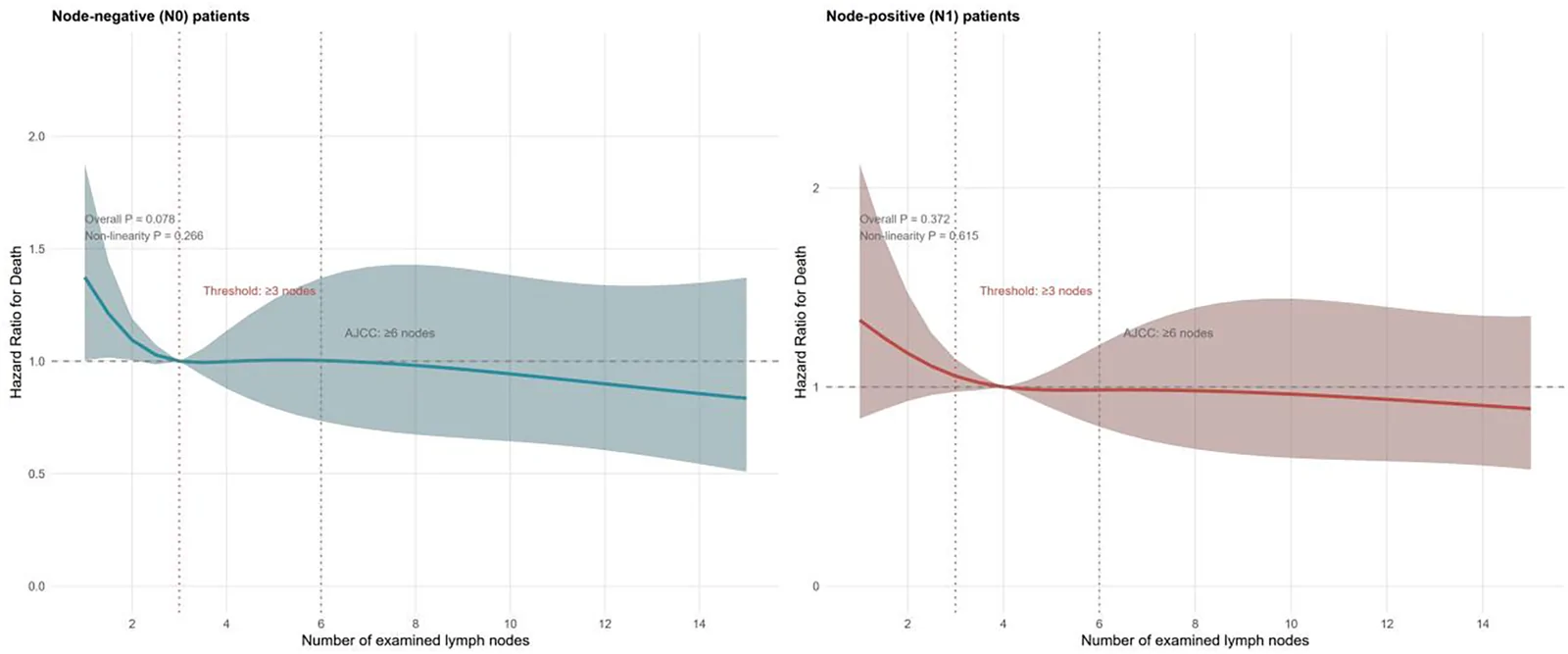
Restricted cubic spline analysis of the association between the number of examined lymph nodes and overall survival (OS), stratified by nodal status. **(A)** N0 patients; **(B)** N1 patients. Four-knot restricted cubic splines were fitted within unadjusted Cox models, treating node count as a continuous variable, with hazard ratios expressed relative to the subgroup median node count (N0: 3 nodes; N1: 4 nodes). Analyses included patients with ≥1 examined node (N0: n = 654; N1: n = 282). N0: overall P = 0.078, non-linearity P = 0.266; N1: overall P = 0.372, non-linearity P = 0.615. Dotted vertical lines mark the ≥3 (red) and ≥6 (gray) thresholds; solid lines represent hazard ratios and shaded bands the 95% confidence intervals.

### 3.6 Sensitivity analysis

To assess the robustness of the primary findings, several sensitivity analyses were performed (**Table 6**). First, using an alternative threshold of ≥3 examined lymph nodes, a significant association with OS was observed (HR 0.85, 95% CI 0.72–1.00; P = 0.044); however, this association was attenuated and no longer significant after excluding patients with no examined nodes (HR 0.85, 95% CI 0.71–1.03; P = 0.098), indicating that it was driven by patients who underwent no nodal evaluation. Second, after excluding 596 patients (38.9%) who had no lymph nodes examined, LNY ≥6 remained not significantly associated with OS (HR 0.88, 95% CI 0.71–1.09; P = 0.251). Third, analyses stratified by AJCC staging era yielded consistent results (6th-edition era: HR 0.87, 95% CI 0.68–1.11; 7th/8th-edition era: HR 0.85, 95% CI 0.59–1.23; interaction P = 0.734), indicating that revisions of the AJCC staging system during the study period did not materially affect our findings. These results indicate that the primary finding is robust to alternative analytical specifications.

**Table 6:** Sensitivity analyses for the association between LNY extent and overall survival.

Sensitivity analysis	HR	95% CI	P value
Primary analysis (multivariable Cox)	0.88	0.71–1.08	0.209
Excluding patients with no nodes examined (n = 936)	0.88	0.71–1.09	0.251
Alternative threshold: ≥3 examined nodes	0.85	0.72–1.00	0.044
≥3 examined nodes, excluding patients with no nodes examined	0.85	0.71–1.03	0.098
6th-edition era (2004–2015)	0.87	0.68–1.11	0.271
7th/8th-edition era (2016–2022)	0.85	0.59–1.23	0.388

## 4 Discussion

Radical surgical resection remains the only potentially curative treatment for intrahepatic cholangiocarcinoma (ICC), yet long-term outcomes after surgery are heterogeneous and are strongly influenced by tumor burden and lymph node involvement. Accurate nodal staging is critical for prognostic stratification and treatment planning, and current AJCC guidelines recommend the examination of at least six lymph nodes to ensure adequate nodal evaluation (16). However, in the context of evolving systemic therapies and ongoing debate about the value of extended lymphadenectomy, whether the number of examined lymph nodes reflects a true therapeutic effect of surgical dissection or primarily serves a staging function remains unresolved (2).

In this population-based SEER study, we reappraised the prognostic role of nodal examination in patients undergoing curative-intent resection for ICC, with explicit attention to the distinction between the number of examined lymph nodes and the surgical extent of lymphadenectomy. In the SEER database, the recorded variable is the count of examined lymph nodes, which reflects a composite of both the anatomical extent of surgical dissection and the thoroughness of pathological processing. Our primary exposure variable should therefore be interpreted as a measure of overall nodal evaluation rather than a direct surrogate for the aggressiveness of lymphadenectomy alone. Because prospective trials are difficult to conduct in this setting and single-center retrospective series are often constrained by follow-up and treatment information (17, 18), we used population-level data and applied multivariable adjustment, inverse probability of treatment weighting (IPTW), and nodal status–stratified analyses to reduce confounding and improve interpretability.

Previous SEER-based studies have yielded heterogeneous conclusions, partly owing to methodological limitations, including insufficient adjustment for chemotherapy, limited use of propensity score–based methods, and pooling of node-negative and node-positive patients without stratification (**Supplementary Table S5**). We therefore designed the present study to address these gaps by applying IPTW, stratifying by nodal status, and formally evaluating the association between LNY and nodal detection, thereby distinguishing the staging contribution of nodal evaluation from an apparent survival benefit.

Across analyses, the results were consistent: in the overall cohort, examining at least six lymph nodes was not independently associated with improved overall survival (OS) after multivariable adjustment, and no survival benefit was observed in either the N0 or N1 subgroups after IPTW weighting. However, these findings should be interpreted with caution. The majority of patients (38.9%) had no lymph nodes examined, which may reflect a cohort in whom lymphadenectomy was particularly limited, potentially diluting any detectable therapeutic effect. Moreover, only 247 patients (16.1%) met the guideline-recommended threshold of ≥6 examined nodes, which limits statistical power in subgroup analyses. Taken together, these results suggest that the clinical value of lymph node evaluation in this cohort was more consistent with a staging function, although a modest therapeutic effect of lymphadenectomy cannot be entirely excluded.

Consistent with prior SEER-based and institutional studies, nodal metastasis (N1) remained among the strongest adverse prognostic factors for OS. Notably, the direction of association for tumor multiplicity was inverted compared with conventional expectation (HR 1.27 for single vs. multiple tumors; 95% CI, 1.08–1.50; P = 0.004), which we interpret as a manifestation of selection bias in surgical cohorts. Importantly, more extensive nodal evaluation was associated with a significantly higher likelihood of identifying N1 disease (OR, 4.06; 95% CI, 2.99–5.52; P < 0.001). This pattern is compatible with a stage-migration explanation: a greater number of examined nodes reduces the probability of missed nodal metastases, leading to more accurate N-classification and, consequently, more favorable observed survival in patients classified as node-negative.

Preoperative imaging has limited accuracy for nodal assessment in ICC (19, 20, 28, 29). Together with our logistic regression findings, these observations reinforce the primary role of nodal evaluation as a staging tool: the improved survival associated with more extensive nodal examination is likely attributable to better risk stratification rather than a direct therapeutic effect of removing metastatic lymph nodes.

In our N0 analyses, unadjusted Kaplan-Meier curves suggested numerically better outcomes with more extensive nodal evaluation, but this association was attenuated after IPTW and was not significant in weighted Cox models. In the N1 subgroup, neither weighted Cox models nor spline analyses showed evidence of a dose–response relationship between nodal evaluation extent and OS. Chemotherapy was consistently associated with better OS, particularly in N1 patients, consistent with prospective adjuvant evidence in biliary tract cancer (21, 22).

The restricted cubic spline (RCS) analysis in N0 patients adds nuance to these findings. The RCS revealed a monotonic decrease in the hazard of death with increasing node counts (overall P = 0.078), with non-linearity testing non-significant (P = 0.266), indicating an approximately log-linear relationship rather than a discrete biological threshold. The ≥3 cut-off remained significantly associated with better survival in N0 patients after multivariable adjustment (in unweighted models) (HR, 0.74; 95% CI, 0.61–0.91; P = 0.004), whereas the ≥6 cut-off was not (HR, 0.77; 95% CI, 0.58–1.04; P = 0.089). This difference is attributable to statistical power (343 vs. 142 patients classified as high-yield at the two thresholds) rather than evidence that three nodes is a superior threshold. The AJCC-recommended threshold of six nodes remains clinically appropriate; these data merely demonstrate that even partial compliance confers measurable staging benefit.

An additional consideration worth noting is the emerging evidence that extensive lymphadenectomy may interact with the efficacy of postoperative immunotherapy. A recent study by Yang et al. found that excessive lymph node dissection (≥36 nodes) was associated with worse outcomes in gastric cancer patients receiving adjuvant immunotherapy (23), potentially reflecting the immunological role of tumor-draining lymph nodes in sustaining anti-tumor immune responses. Although this evidence is preliminary and derived from a different tumor type, it raises the question of whether the risk–benefit balance of extensive lymphadenectomy may need to be reconsidered as immune checkpoint inhibitors become more widely used in ICC (24, 25). Further research is needed to clarify this relationship in biliary tract cancers.

Several limitations should be acknowledged. First, as a retrospective SEER-based analysis, selection bias and residual confounding cannot be fully excluded. Second, the number of examined lymph nodes reflects both surgical and pathological factors, and SEER does not distinguish between these components. Third, SEER lacks recurrence patterns, performance status, comorbidity, and anatomical extent of lymphadenectomy data. Moreover, SEER does not distinguish between the small-duct (peripheral) and large-duct (periductal infiltrating) histologic subtypes of ICC, which differ in lymph node metastasis frequency and prognosis, potentially confounding the observed association between LNY and survival. Fourth, the low proportion of patients (16.1%) with ≥6 examined nodes limits generalizability and statistical power. Fifth, the SEER cause-of-death variable, while validated, may have some misclassification. Finally, recent reports suggesting survival advantages of lymphadenectomy in selected node-negative patients (5, 26) highlight the need for prospective data to reconcile these differences.

Notwithstanding these limitations, our findings support current AJCC recommendations for adequate nodal evaluation in ICC, while recognizing that this standard is achieved in a limited proportion of patients in routine practice. The primary value of lymph node examination in this study appeared to be staging adequacy; improvements in long-term outcomes are more likely to depend on effective systemic therapy and multidisciplinary management, particularly for node-positive patients. Future prospective studies with standardized nodal assessment protocols and comprehensive treatment data are warranted to further define the optimal role of lymphadenectomy in the evolving therapeutic landscape of ICC.

In summary, in this population-based cohort of resected ICC, more extensive nodal evaluation was not independently associated with improved OS after adjustment. These findings are consistent with a staging benefit of nodal evaluation, though a modest therapeutic effect cannot be excluded. The emerging concern that extensive lymphadenectomy may carry immunological costs in the context of immunotherapy (23) warrants further investigation. These data suggest that achieving adequate nodal staging should be prioritized, while recognizing that the optimal balance between staging accuracy and surgical extent may evolve as systemic therapies continue to advance.

## 5 Conclusion

This population-based analysis of resected ICC patients suggests that the number of examined lymph nodes was not independently associated with improved OS after multivariable adjustment. These findings are consistent with a staging benefit of nodal evaluation, though the observational nature of the data precludes definitive conclusions about the presence or absence of a therapeutic effect. The potential immunological implications of extensive lymphadenectomy in the context of emerging immunotherapy regimens warrant further investigation. Prospective studies are needed to clarify the optimal role of lymph node evaluation in the evolving therapeutic landscape of ICC.

## Data Availability

The data analysed in this study are publicly available in the SEER database (SEER Research Data, 17 Registries, November 2024 submission, released April 2025; case listing generated 23 September 2025; https://seer.cancer.gov). Further inquiries can be directed to the corresponding author.
