# Supplementary material for: Number of examined lymph nodes and overall survival in intrahepatic cholangiocarcinoma: a population-based study suggesting a staging benefit
Source: Front Oncol. 2026 Sep 28;16:1925253. doi: 10.3389/fonc.2026.1925253 (PMC13655046; doi:10.3389/fonc.2026.1925253)
Supplement: Supplementary file 1 [file SupplementaryFile1.docx]

*Figure S1. Kaplan–Meier estimates of overall survival according to lymph node yield (0, 1–5, ≥6 examined nodes) in the overall cohort (n = 1,532). Log-rank P = 0.444. Dashed lines indicate median survival; numbers at risk are shown below.*

*Figure S2. Kaplan–Meier estimates of overall survival according to lymph node yield in node-negative (N0) patients (n = 1,238). Log-rank P = 0.033.*

*Figure S3. Kaplan–Meier estimates of overall survival according to lymph node yield in node-positive (N1) patients (n = 294). Log-rank P = 0.713.*

*Figure S4. Love plot showing standardized mean differences (SMD) of covariates across the three LNY groups before (grey) and after (red) multinomial generalized propensity score IPTW. The dashed vertical line indicates the balance threshold of |SMD| < 0.10.*

*Figure S5. IPTW-weighted Kaplan–Meier estimates of overall survival according to lymph node yield group in the overall cohort.*

*Table S1. Baseline characteristics of patients with resected intrahepatic cholangiocarcinoma, stratified by number of examined lymph nodes (0, 1–5, ≥6).*

| Variable | Level | 0 (n=596) | 1-5 (n=689) | >=6 (n=247) | P |
| --- | --- | --- | --- | --- | --- |
| Age | ＜60 | 141 (23.7%) | 214 (31.1%) | 84 (34.0%) | 0.002 |
|  | ≥60 | 455 (76.3%) | 475 (68.9%) | 163 (66.0%) |  |
| Sex | Female | 271 (45.5%) | 403 (58.5%) | 143 (57.9%) | <0.001 |
|  | Male | 325 (54.5%) | 286 (41.5%) | 104 (42.1%) |  |
| Race | White | 443 (74.3%) | 562 (81.6%) | 194 (78.5%) | 0.007 |
|  | Non-white | 153 (25.7%) | 127 (18.4%) | 53 (21.5%) |  |
| Tumor number | Single | 390 (65.4%) | 494 (71.7%) | 181 (73.3%) | 0.019 |
|  | Multiple | 206 (34.6%) | 195 (28.3%) | 66 (26.7%) |  |
| Histological grade | I+II | 403 (67.6%) | 485 (70.4%) | 169 (68.4%) | 0.550 |
|  | III+IV | 193 (32.4%) | 204 (29.6%) | 78 (31.6%) |  |
| T category | T1+T2 | 514 (86.2%) | 544 (79.0%) | 180 (72.9%) | <0.001 |
|  | T3+T4 | 82 (13.8%) | 145 (21.0%) | 67 (27.1%) |  |
| N stage | N0 | 584 (98.0%) | 512 (74.3%) | 142 (57.5%) | <0.001 |
|  | N1 | 12 (2.0%) | 177 (25.7%) | 105 (42.5%) |  |
| Chemotherapy | No | 342 (57.4%) | 295 (42.8%) | 96 (38.9%) | <0.001 |
|  | Yes | 254 (42.6%) | 394 (57.2%) | 151 (61.1%) |  |
| Radiotherapy | No | 537 (90.1%) | 592 (85.9%) | 204 (82.6%) | 0.007 |
|  | Yes | 59 (9.9%) | 97 (14.1%) | 43 (17.4%) |  |
| Year of diagnosis | 2007-2010 | 113 (19.0%) | 94 (13.6%) | 29 (11.7%) | 0.025 |
|  | 2011-2014 | 151 (25.3%) | 164 (23.8%) | 53 (21.5%) |  |
|  | 2015-2018 | 103 (17.3%) | 128 (18.6%) | 54 (21.9%) |  |
|  | 2019-2022 | 229 (38.4%) | 303 (44.0%) | 111 (44.9%) |  |
| Tumor size, mm | mean ± SD | 52.5 ± 31.9 | 56.1 ± 32.8 | 53.6 ± 32.3 | 0.079 |
|  | median (IQR) | 45 (30–65) | 50 (32–75) | 47 (30–71) |  |

*Table S2. Multivariable Cox proportional hazards model for overall survival in the overall cohort (three-level LNY).*

| Comparison | HR | 95% CI | P |
| --- | --- | --- | --- |
| LNY 1–5 vs 0 | 0.91 | 0.77–1.07 | 0.249 |
| LNY ≥6 vs 0 | 0.82 | 0.65–1.04 | 0.099 |
| LNY ≥6 vs 1–5 | 0.91 | 0.73–1.12 | 0.357 |
| P for trend | 0.91 | 0.81–1.02 | 0.090 |

*Table S3. Multivariable Cox proportional hazards model for overall survival in node-negative (N0) patients.*

| Comparison | HR | 95% CI | P |
| --- | --- | --- | --- |
| LNY 1–5 vs 0 | 0.84 | 0.70–1.01 | 0.069 |
| LNY ≥6 vs 0 | 0.70 | 0.52–0.96 | 0.025 |
| P for trend | 0.84 | 0.74–0.96 | 0.010 |

*Table S4. Multivariable Cox proportional hazards model for overall survival in node-positive (N1) patients.*

| Comparison | HR | 95% CI | P |
| --- | --- | --- | --- |
| LNY 1–5 vs 0 | 1.85 | 0.92–3.74 | 0.086 |
| LNY ≥6 vs 0 | 1.73 | 0.83–3.59 | 0.145 |
| P for trend | 1.07 | 0.83–1.37 | 0.612 |

*Table S5. Multivariable logistic regression for predictors of nodal metastasis (N1 vs N0).*

| Comparison | OR | 95% CI | P |
| --- | --- | --- | --- |
| LNY 1–5 vs 0 | 16.29 | 8.91–29.78 | <0.001 |
| LNY ≥6 vs 0 | 34.00 | 18.01–64.18 | <0.001 |
| LNY ≥6 vs 1–5 | 2.09 | 1.52–2.86 | <0.001 |

*Table S6. Covariate balance before and after multinomial GPS-based IPTW (maximum standardized mean difference across treatment pairs; threshold |SMD| < 0.10). Effective sample sizes after weighting: 0 = 226, 1–5 = 649, ≥6 = 184.*

| Covariate | SMD unweighted | SMD weighted |
| --- | --- | --- |
| Age ≥60 yr | 0.104 | 0.010 |
| Male sex | 0.130 | 0.042 |
| Tumor size (mm) | 0.109 | 0.154 |
| Multiple tumours | 0.078 | 0.032 |
| T category T3–T4 | 0.134 | 0.009 |
| Grade III–IV | 0.028 | 0.053 |
| Year 2007–2010 | 0.072 | 0.038 |
| Year 2011–2014 | 0.039 | 0.058 |
| Year 2015–2018 | 0.046 | 0.038 |
| Year 2019–2022 | 0.065 | 0.067 |
| N stage N1 | 0.405 | 0.009 |

*Table S7. IPTW-weighted multivariable Cox proportional hazards model for overall survival in the overall cohort (three-level LNY, robust standard errors).*

| Comparison | HR | 95% CI | P |
| --- | --- | --- | --- |
| LNY 1–5 vs 0 | 0.98 | 0.80–1.19 | 0.834 |
| LNY ≥6 vs 0 | 0.90 | 0.69–1.18 | 0.457 |
| LNY ≥6 vs 1–5 | 0.92 | 0.72–1.18 | 0.515 |
| P for trend | 0.96 | 0.84–1.09 | <0.001 |
