# Supplementary material for: Number of examined lymph nodes and overall survival in intrahepatic cholangiocarcinoma: a population-based study suggesting a staging benefit
Source: Front Oncol. 2026 Sep 28;16:1925253. doi: 10.3389/fonc.2026.1925253 (PMC13655046; doi:10.3389/fonc.2026.1925253)
Supplement: Supplementary file 2 [file Table1.docx]

**Supplementary Material**

**Table S1A. SEER case selection criteria.**

| **SEER Variable** | **Coding** |
| --- | --- |
| Site and Morphology — Site recode ICD-O-3 | Intrahepatic Bile Duct |
| Year of diagnosis | 2004–2022 |
| Age at diagnosis | ≥18 years |
| Surgery performed | Yes (RX Summ—Surg Prim Site codes 20–90) |
| Lymph node examination | 0–26 nodes examined |
| Survival data | Complete |

**Table S1B. SEER variables and coding used in this study.**

| **Category** | **SEER Variable** | **Coding** |
| --- | --- | --- |
| Lymph node surgery | RX Summ—Scope Reg LN Sur (2003+) | None; 1–3 nodes; ≥4 nodes removed |
| Lymph nodes examined | RX Summ—Scope Reg LN Sur (2003+) | Number of regional lymph nodes examined |
| Tumour size | Tumour size summary | Continuous (mm) |
| Histological grade | Grade | I–IV |
| AJCC stage | AJCC stage (7th/8th ed) | I–IV |
| T category | Derived AJCC T | T1–T4 |
| N category | Derived AJCC N | N0–N1 |
| Survival months | Survival months | Continuous |
| Vital status | Vital status recode | Dead; Alive |
| Chemotherapy | Chemotherapy recode | Yes; No/Unknown |
| Radiotherapy | Radiation recode | Yes; No/Unknown |
| Marital status | Marital status at diagnosis | Married; Single; Other |

**Table S2A. International Classification of Disease, 3rd edition (ICD-O-3) codes defining the cohort of patients with intrahepatic cholangiocarcinoma.**

| **Topography** | **Histology** |
| --- | --- |
| C22.0 (Liver) | 8160/3, 8161/3 |
| C22.1 (Intrahepatic bile duct) | 8140/3, 8141/3, 8160/3, 8161/3, 8260/3, 8310/3, 8480/3, 8481/3, 8500/3 |

**Table S2B. Surveillance, Epidemiology, and End Results (SEER) program 2003 liver and intrahepatic bile duct site-specific surgery codes.**

| **Code** | **Description** |
| --- | --- |
| 20 | Wedge or segmental resection, NOS |
| 21 | Wedge resection |
| 22 | Segmental resection |
| 25 | Lobectomy |
| 30 | Extended lobectomy |
| 36 | Bisegmentectomy |
| 37 | Trisegmentectomy |
| 40 | Hepatectomy, NOS |
| 50 | Liver transplant |
| 55 | Transplantation |
| 60 | Radical surgery |
| 70 | Surgery, NOS |
| 90 | Surgery, unknown type |

Software: Surveillance Research Program, National Cancer Cancer Institute SEER*Stat software (www.seer.cancer.gov/seerstat) version 9.0.41.4.

**Table S3. Comparison of SEER-Based Studies on Lymph Node Dissection in ICC**

| Year / First author | Endpoint | PSM | IPTW | Subgroup analysis | Chemo adjusted | Main conclusion | LND–prognosis | Interpretation |
| --- | --- | --- | --- | --- | --- | --- | --- | --- |
| 2011 – Clark | OS | No | No | No | No | LN status predicts OS; LND underused | Not evaluated | Staging |
| 2016 – Vitale | OS | Yes | No | Limited | Unclear | No OS benefit after matching | No survival benefit | Therapeutic |
| 2018– Zhang | OS | No | No | No | No | LND not associated with OS | No survival benefit | Therapeutic |
| 2021 – Hu | OS | Yes | No | No | Unclear | LND group showed worse OS | Worse survival observed | Therapeutic |
| 2023 – Zhu | OS / CSS | Yes | No | Yes | Yes | No OS/CSS benefit; ≥6 improves staging | No survival benefit | Staging |
| 2023–Zhang | OS | No | No | Yes | No | LND effect varies by side | Heterogeneous effect | Heterogeneity |
| 2025 – Ou | CSS | No | No | No | Unclear | Higher LN count improves CSS | Survival benefit reported | Therapeutic |
